# Oxygen content-related DNA damage of graphene oxide on human retinal pigment epithelium cells

**DOI:** 10.1007/s10856-021-06491-0

**Published:** 2021-02-27

**Authors:** Liling Ou, Xiujuan Lv, Zixia Wu, Weibo Xia, Yida Huang, Luya Chen, Wenjie Sun, Yao Qi, Mei Yang, Lei Qi

**Affiliations:** 1 State Key Laboratory of Ophthalmology, Optometry and Visual Science, Institute of Advanced Materials for Nano-Bio Applications, School of Ophthalmology and Optometry, School of Biomedical Engineering, Wenzhou Medical University, Wenzhou, China; 2 Department of Ultrasonic, The First Hospital of Qiqihar, Affiliated Qiqihar Hospital, Southern Medical University, Qiqihar, China

## Abstract

Arguments regarding the biocompatibility of graphene-based materials (GBMs) have never ceased. Particularly, the genotoxicity (e.g., DNA damage) of GBMs has been considered the greatest risk to healthy cells. Detailed genotoxicity studies of GBMs are necessary and essential. Herein, we present our recent studies on the genotoxicity of most widely used GBMs such as graphene oxide (GO) and the chemically reduced graphene oxide (RGO) toward human retinal pigment epithelium (RPE) cells. The genotoxicity of GO and RGOs against ARPE-19 (a typical RPE cell line) cells was investigated using the alkaline comet assay, the expression level of phosphorylated p53 determined via Western blots, and the release level of reactive oxygen species (ROS). Our results suggested that both GO and RGOs induced ROS-dependent DNA damage. However, the DNA damage was enhanced following the reduction of the saturated C–O bonds in GO, suggesting that surface oxygen-containing groups played essential roles in the reduced genotoxicity of graphene and had the potential possibility to reduce the toxicity of GBMs via chemical modification.

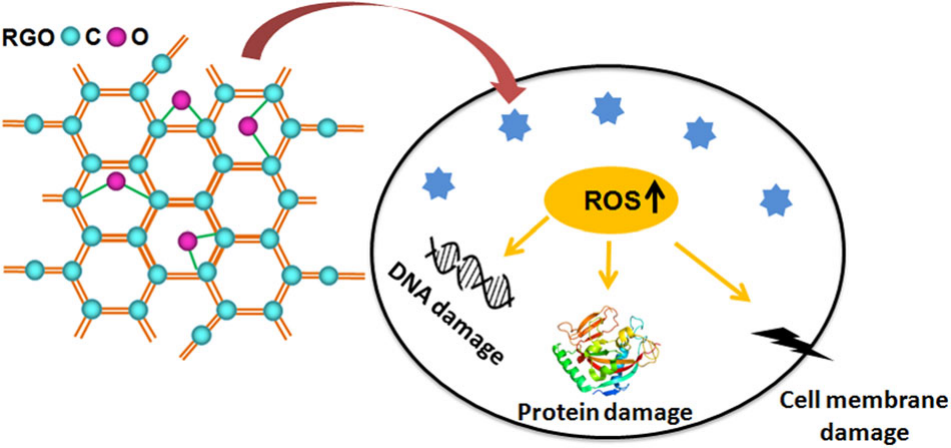

## Introduction

Graphene-based materials (GBMs) possess a large active surface area for substance transportation by taking advantage because of their unique one-atom thickness and two-dimensional plane, which are favorable for applications in areas of disease therapy and diagnostics such as biosensing, drug delivery, and tissue engineering [1–3]. Graphene oxide (GO) which is the most important initial molecular unit of the GBMs, is generally thought to be compatible for biological applications due to its hydrophilic functional groups [4]. Similar to other research groups’ works on cytotoxicity of GO [5, 6], we have found out that oxygen-containing functional groups modified graphene prepared from both chemical oxidation and edge-functionalized ball milling method exhibited good biocompatibility with ocular cells and tissues [7, 8]. However, despite of numerous efforts, no accordance has been reached regarding the safety of GO, in considering the size-, concentration dependent or various cell strains [8–11]. Moreover, the oxidation degree of the GO is also an inevitable indicator of the safety of GO on biological organisms.

A number of studies have focused on the biological effects of GO and reduced GO (RGO) on mammalian cells [12, 13]. Two distinct points have been proposed. In the Das et al. study, RGOs that were reduced by the hydrazine hydrate method, were found to be safer than GO on human umbilical vein endothelial cells (HUVECs). They hypothesized that more reactive functional groups (e.g., –OH, –COOH) of GO potential to interact with the macromolecules than in RGO and enhancing the bio-nano interaction dependent toxicity [12]. However, Zhang et al. prepared RGOs by reducing the amount of KMnO_4_ used during hummer’s method. These RGOs exhibited a higher degree of cytotoxicity and apoptosis as the degree of oxidation decreased. They found that the less oxidized GO produced a higher level of reactive oxygen species (ROS) and induced the oxidative damage [13]. Researchers have also found that both GO and RGO solid substrates are compatible with NIH-3T3 fibroblast [14]. This discrepancy inspired us to investigate the mechanisms of oxidation-state dependent cytotoxicity of GO.

In addition, GO is readily transported across the cell membrane [7], because of its unique size and morphology, resulting in the destruction of biomolecules such as nucleic acids, lipids, and proteins. This may cause unpredictable genotoxicity of GO which has also attracted intense attention. Though we have reported mild genotoxicity against human retinal pigment epithelium (RPE) cells for hydroxyl-functionalized graphene (G-OH) at the concentration up to 100 μg/mL, which synthesized by ball milling [7]. Detailed genotoxicity studies of GOs with different oxidation-states on ocular cells, such as RPE cells are needed and worthwhile since the risk of eye contamination by GO for people, who work in the fields of graphene production, transportation, research, and biomedical applications, is very high. In this work, RGOs were prepared by Na-citrate reduction and the RGOs with different oxidation states were obtained by controlling the reduction time. GO displayed much better cytotoxicity than RGOs. Furthermore, we measured the genotoxicity of GO compared to RGOs against ARPE-19 cells by means of alkaline Comet assay for DNA damage, Western blotting for the detection of phosphorylated p53 expression, and ROS generation. Though ROS-dependent DNA damage caused by both GO and RGOs was observed, the genotoxicity of the RGOs was much stronger than that of GO. The lower oxygen content led to stronger genotoxicity, suggesting a possible relationship between the oxygen content and genotoxicity of GBMs.

## Materials and methods

### Preparation of graphene oxide (GO)

GO was prepared via a modified Hummer’s method. Typically, graphite was oxidized in mixed oxidant of H_2_SO_4_, NaNO_3_, KMnO_4_, and 30% (w/w) H_2_O_2_ for 2 h. The solid mixture was then collected after centrifugation. A mixed solution of 3% (w/w) H_2_SO_4_/0.5% (w/w) H_2_O_2_ was then used to wash the mixture and exfoliate the graphite oxide using ultrasonication for 30 min. DI water was further used to rinse and exfoliate the nanosheets via ultrasonication over 2 h.

### Preparation of reduced graphene oxide (RGO)

RGOs were prepared via a “green” reduction approach with the use of Na-citrate. Typically, 0.05 mg Na-citrate was added to the GO dispersion (25 µg/mL) with a final volume of 40 mL. The reactions were carried out in polytetrafluoroethylene reaction kettles at 100 °C for 3, 6, 9, and 12 h. The corresponding reduced products were named RGO-3, RGO-6, RGO-9, and RGO-12. The resulting samples were then centrifuged at 8000 rpm for 10 min. The as-synthesized supernatants were subsequently transferred into D.I. H_2_O until the pH value was to 5.5. Another of 30 min ultrasonication was produced at 100 W just before the biocompatibility assays.

### Characterization

The GO and RGO samples were characterized using UV–visible spectrophotometry (UV–Vis, Agilent Cary 100), Fourier transform infrared spectroscopy (FTIR, Thermo Nicolet 6700), X-ray photoelectron spectroscopy (XPS), transmission electron microscopy (TEM), and atomic force microscopy (AFM).

### Cell culture

Human RPE cells (ARPE-19) were cultured in DMEM/F12 (Gibco) supplemented with 10% (v/v) qualified Fetal Bovine Serum (Gibco) and gentamicin (50 µg/mL, Gibco) at 37 °C with 10% CO_2_.

### Cell viability

Cell Counting Kit-8 (CCK-8, Dojindo) was used to determine the cell viability. The cells were seeded in 96-well plates at a density of 3000 cells/well and preincubated for 24 h. The cells were cultured with GO or RGOs at various concentrations (10, 50, 100, 200 µg/mL). Cells cultured in medium without the addition of GO or RGOs were used as the negative control. After 6, 12, 24, 48, and 72 h of incubation, the culture medium was replaced with 100 µL of CCK-8 solution diluted in culture medium at a ratio of 1:10 (V/V) and incubated for an additional 3 h at 37°C. The optical density (OD) of each well at 450 nm was recorded by means of a SpectraMax M5 (Molecular Devices). The cell viability was expressed as the percentage of (OD_test_ − OD_blank_)/(OD_negative_ − OD_blank_), where OD_blank_ is the optical density of culture medium without ARPE-19 cells.

Calcein-AM/PI staining (Dojindo) was further used for the direct observation of living/dead cells. The cells were cultured in 24-well plates at a density of 30,000 cells per well. After 24 h, the cell medium was changed to fresh medium with GO or RGOs (200 μg/mL) for 72 h. The cells were dyed with the staining solution for 15 min and observed under a fluorescence microscope (OLYMPUS IX81) with green/red fluorescent exciters (Ex/Em of Calcein-AM: 490/515; Ex/Em of PI: 535/617).

### Reactive oxygen species (ROS) assay

The cells were seeded in 96-well black plates (Clear bottom, Costar 3603) at 10,000 cells per well and preincubated for 24 h. The cells were cultured with various concentrations of GO or RGOs (10, 50, 100, 200 µg/mL) for 6, 12, 24, 48, and 72 h. The oxidant-sensitive dye DCFH-DA was used for ROS detection (Reactive Oxygen Species Assay Kit, Beyotime Institute of Biotechnology, China). The culture medium for all cells were replaced by 100 µL of new culture medium (without supplementation of Fetal Bovine Serum) containing 10 µM DCFH-DA for 20 min at 37 °C in the dark. The cells were washed with culture medium (without supplementation of Fetal Bovine Serum) for three times. The positive control was prepared by culturing normal cells with culture medium containing 500 mM H_2_O_2_ for 30 min after the cells were labeled by DCFH-DA, while the negative control was the normal culture medium without the addition of nanomaterials. The fluorescence intensity was detected by means of a SpectraMax M5 (Molecular Devices) with an excitation wavelength of 488 nm and an emission wavelength at 525 nm in the mode of bottom read mode. The ROS level was expressed as the ratio of (*I*_test_ − *I*_blank_)/(*I*_negative_ − *I*_blank_), where *I*_blank_ is the fluorescence intensity of the culture medium without the cells. The cells incubated with GO or RGOs at 200 µg/mL for 72 h were further observed by fluorescence microsccopy with green fluorescent excitation.

### Alkaline Comet assay

An alkaline comet assay reagent kit (TREVIGEN) was used to evaluate DNA damage, including single- and double-stranded breaks in cells. The cells were grown on polystyrene six-well tissue culture plates (Corning) at a density of 200,000 cells per well and preincubated for 24 h. The cells were continuously cultured in an incubator with 100 µg/mL of GO or RGOs for 24 h. Cells cultured in the medium with 0.5 M H_2_O_2_ for 10 min were used as the positive control, while cells incubated without the addition of nanomaterials were used as the negative controls [15]. DNA was visualized by staining with the fluorescent DNA binding dye PI and observed by a fluorescence microscope (OLYMPUS, IX81). The percent of DNA in the tail was calculated by the software of Comet Assay Software Project.

### Western blot

The cells were grown on polystyrene six-well tissue culture plates at a density of 2.5 × 10^5^ cells/well and preincubated for 24 h. The cells were continuously cultured with the addition of GO or RGOs (100 µg/mL) for 24 h. Cells cultured in the medium with no addition of nanomaterials were used as the negative control. The cells were harvested and lysed in western cracking buffer (Beyotime) for 5 min. The total protein solutions were collected by centrifugation at 14,000 rpm for 15 min and concentrated by an Amicon ultra centrifugal filter (Millipore, 0.5 mL, 10 kDa) at 4 °C. The concentrations of protein were determined by the BCA Protein Assay Kit (Thermo). Western blotting was used to analyze the phosphorylated p53 (pi-p53) expression level by probing with a phosphorylated p53-Ser15 rabbit polyclonal antibody (Abcam). The samples were blotted with Goat Anti-Rabbit IgG H&L (HRP) secondary antibodies (Abcam) after incubation with primary antibody. The GAPDH was used as the loading control probe with a HRP-anti-GAPDH monoclonal antibody (KANGCHEN). A chemiluminescent substrate kit (SuperSignal West Dura Extended Duration Substrate, Thermo) was used for HRP detection on the immunoblots. The relative band intensities (according to the band of GAPDH) were calculated by the software of Image Lab (Bio-Rad Laboratories).

### Statistical analysis

Six parallel tests were conducted for each practical type. Significance was calculated using unpaired two-tailed Student’s t-test with unequal variance used for statistical analysis. “*” denotes statistical significance (*≤0.05, **≤0.01, ***≤0.001) vs. the control.

## Results

### Characterization of RGOs

**Fig. 1:**
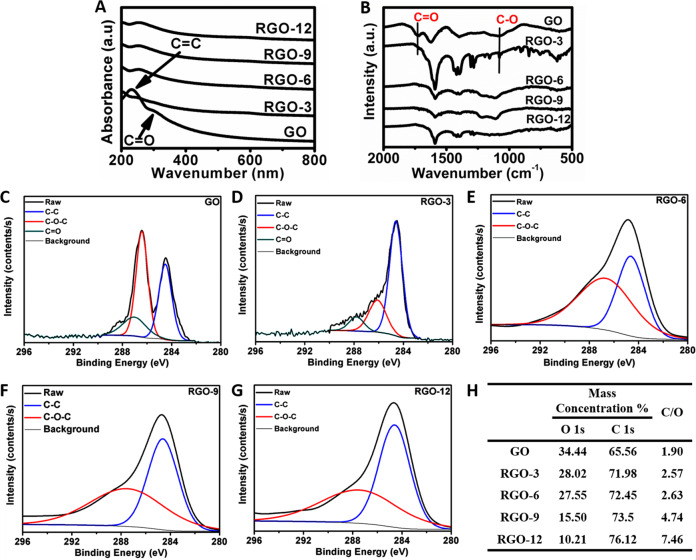
Characterization results of RGOs compared to GO using **A** UV–Vis spectra and **B** FTIR spectra. The XPS high-resolution C1s spectrum of GO (**C**), RGO-3 (**D**), RGO-6 (**E**), RGO-9 (**F**), RGO-12 (**G**) and the mass ratio of carbon to oxygen (C/O) in the GO and RGOs determined by XPS (**H**)

Biocompatible Na-citrate was used as the reductant for the preparation of RGOs to minimize the influence of the reductant on the biocompatibility of RGOs. The reduction of GO was characterized by using different spectroscopies such as UV–Vis, FTIR, and XPS. Two characteristic peaks of GO were observed in the UV–Vis spectra, corresponding to C = C (231 nm) and C = O (300 nm) (Fig. 1A). A bathochromic shift of the peak at 231 nm and the disappearance of the band at 300 nm were observed in the spectrum of sample RGO-3, suggesting the successful reduction of C = O. Similar UV–Vis results were observed for samples RGO-6, RGO-9, and RGO-12, confirming the reduction in oxygen contents in the RGOs. In the FTIR spectra (Fig. 1B), two typical peaks at 1726 cm^−^^1^ and 1081 cm^−1^, attributed to the C = O and C–O, were not present in the spectra of RGO-3, RGO-6, RGO-9, and RGO-12, which was consistent with the UV–Vis measurements. The oxidation states were further confirmed by XPS (Fig. 1C–H), where the relative intensities of the C–O (286.5 eV) and C = O (287.1 eV) peaks decreased in the spectra of the RGOs versus that of GO. As shown in Fig. 1H, the mass ratio of carbon to oxygen (C/O) for the RGOs decreased with increasing reduction time.

**Fig. 2:**
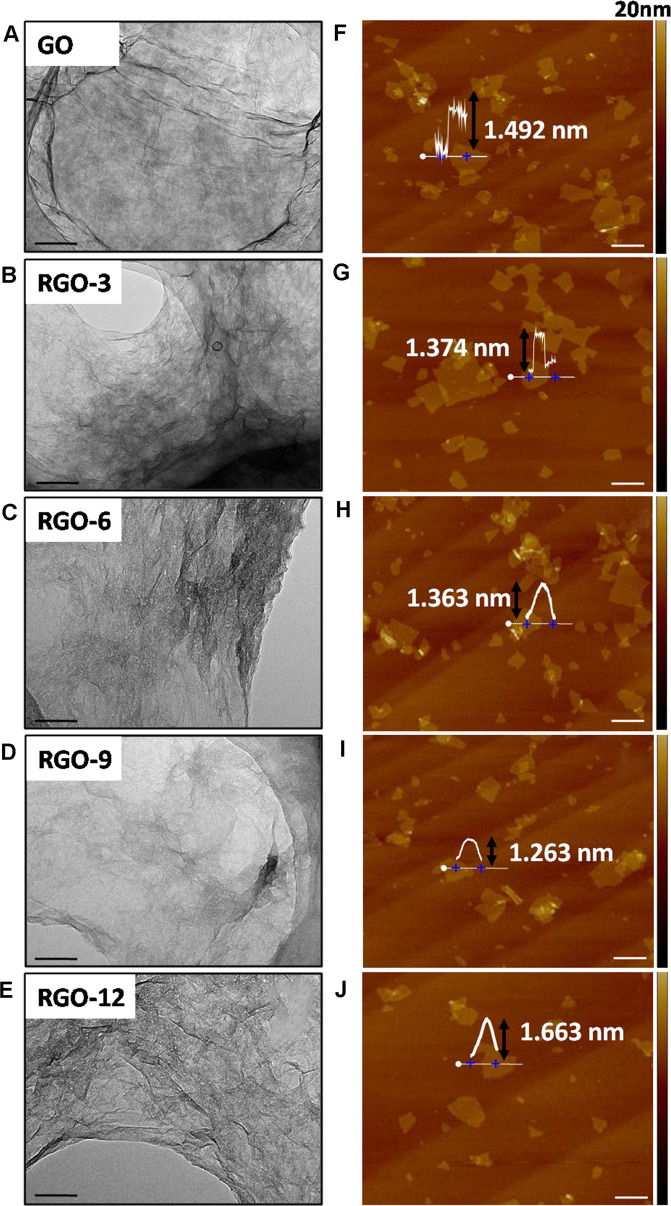
TEM micrographs of GO and RGOs ((**A**–**E**), the scales were 100 nm); AFM micrographs of GO and RGOs ((**F**–**J**), the scales were 1 μm)

The morphologies of GO and RGOs were measured by using TEM and AFM (Fig. 2). Typical transparent, flake-shaped nanosheets were observed in the TEM images of GO and RGOs (Fig. 2A–E). The corresponding AFM images exhibited an average thickness of ~1.4 ± 0.2 nm for GO and RGOs (Fig. 2F–J), suggesting that chemical reduction of oxygen-containing functional groups caused few changes in the thickness and size of graphene, which are generally related to the genotoxicity of graphene derivatives.

### Cell viability

**Fig. 3:**
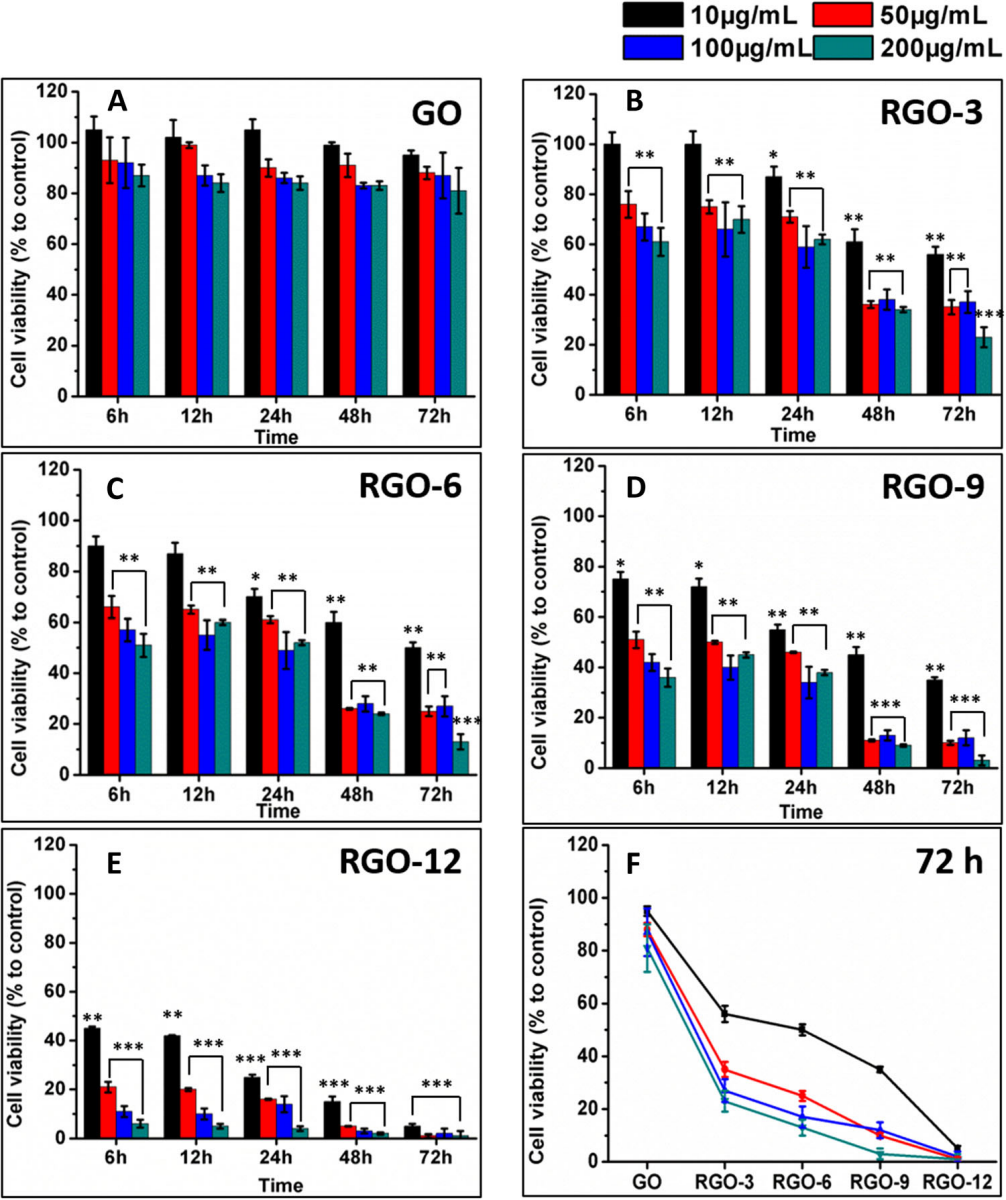
Cell survival rates of ARPE-19 cells incubated with various amounts of GO (**A**), RGO-3 (**B**), RGO-6 (**C**), RGO-9 (**D**), and RGO-12 (**E**) for 6, 12, 24, 48, and 72 h, respectively; **F** the summary of GO and RGOs incubated cells viability after 72 h

The viability of ARPE-19 cells in the presence of GO or RGOs in the culture medium was determined by detecting the activities of intracellular dehydrogenases (CCK-8 assay). The cell viability of ARPE-19 cells with the addition of GO (up to 200 μg/mL) was higher than 80%, even after incubation for 72 h (Fig. 3A). However, the survival rates of ARPE-19 cells were significantly decreased with the reduction of GO to RGOs (Fig. 3B–E). Moreover, the RGOs-induced cell viability declined with increasing RGOs concentrations and culture times. Less than 10% cell viability was observed for the RGO-12 incubated cells after 72 h. The cytotoxicity of GO and RGOs at different concentrations after 72 h of incubation is summarized in Fig. 3F, indicating the strong cytotoxicity of RGOs but the mild cytotoxicity of GO. The viability of ARPE-19 cells was further examined by live/dead staining assay (Fig. S1). ARPE-19 cells were incubated with 200 μg/mL GO or RGOs for 72 h. Compared to the negative control, nearly 50% of cells died after incubation with GO, and over 80% of cells were killed by RGOs. These results confirmed the cell viability observed by the CCK-8 assay.

### ROS level

**Fig. 4:**
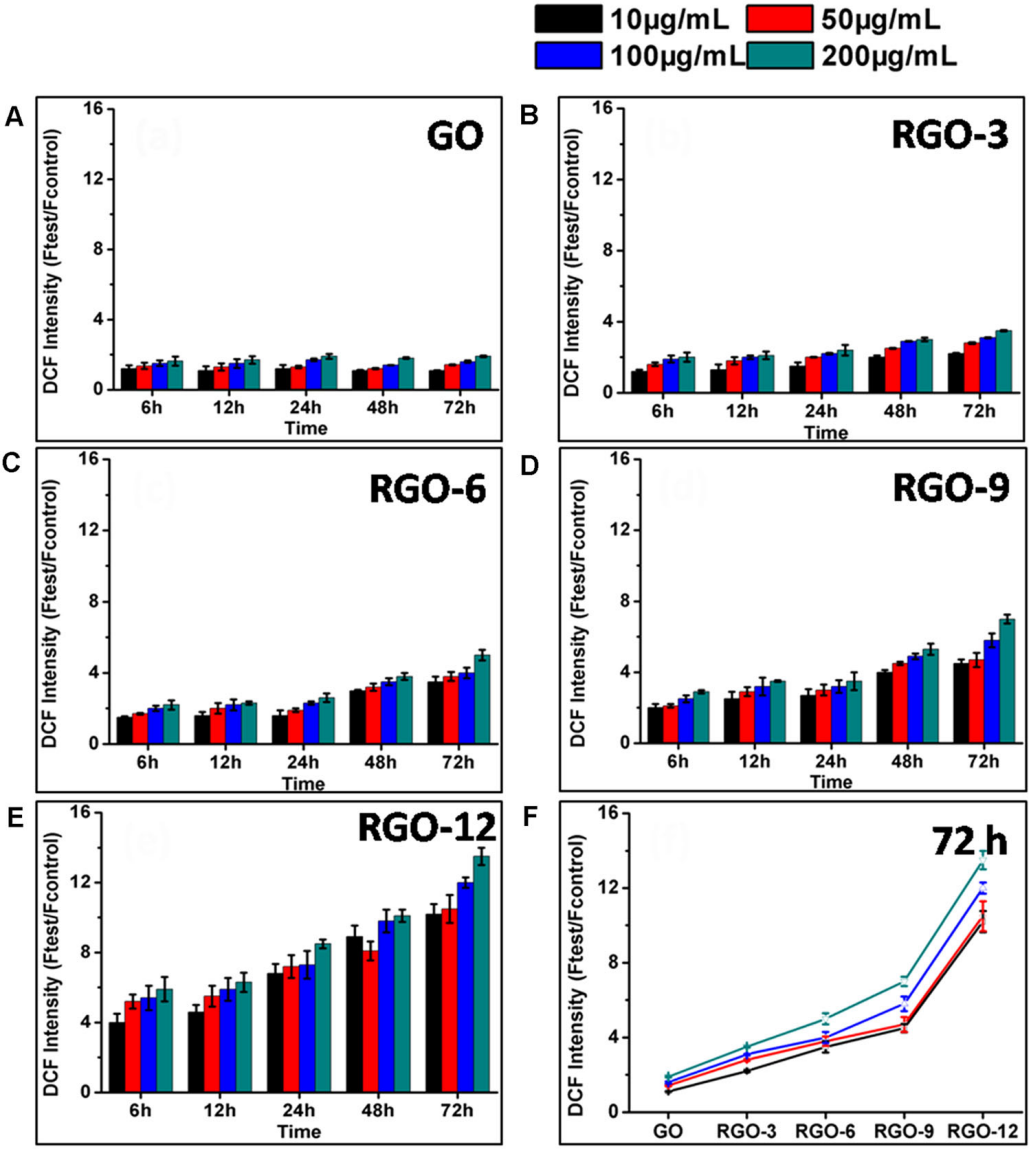
ROS levels of ARPE-19 cells induced by various amounts of GO (**A**), RGO-3 (**B**), RGO-6 (**C**), RGO-9 (**D**), RGO-12 (**E**) after different time and the summary of GO and RGOs incubated cells ROS levels after 72 h (**F**)

The cytotoxicity of carbon nanomaterials is always induced by oxidative stress, arising from ROS-dependent or ROS-independent pathways [16]. In a typical ROS-dependent pathway, the release of ROS is induced, resulting in oxidative stress. In a ROS-independent pathway, however, some materials (e.g., C60) could disturb or oxidize certain components in the cells, leading to the oxidative stress [17]. The ROS levels in ARPE-19 cells induced by GO or RGOs were therefore measured via the DCFH-DA method. As shown in Fig. 4, the ROS levels in ARPE-19 cells were significantly enhanced after incubation with RGOs, and the level of ROS production was directly correlated with the concentrations of RGOs and exposure time. In addition, the lower content of oxygen groups induced a greater ROS increase, which is consistent with the cell viability results. Therefore, the removal of oxygen groups from GO induced the ROS-dependent oxidative stress. The intracellular ROS level was also observed by fluorescence microscopy. As shown in Fig. S2, the intracellular ROS level was significantly increased by RGOs incubation.

### DNA damage

**Fig. 5:**
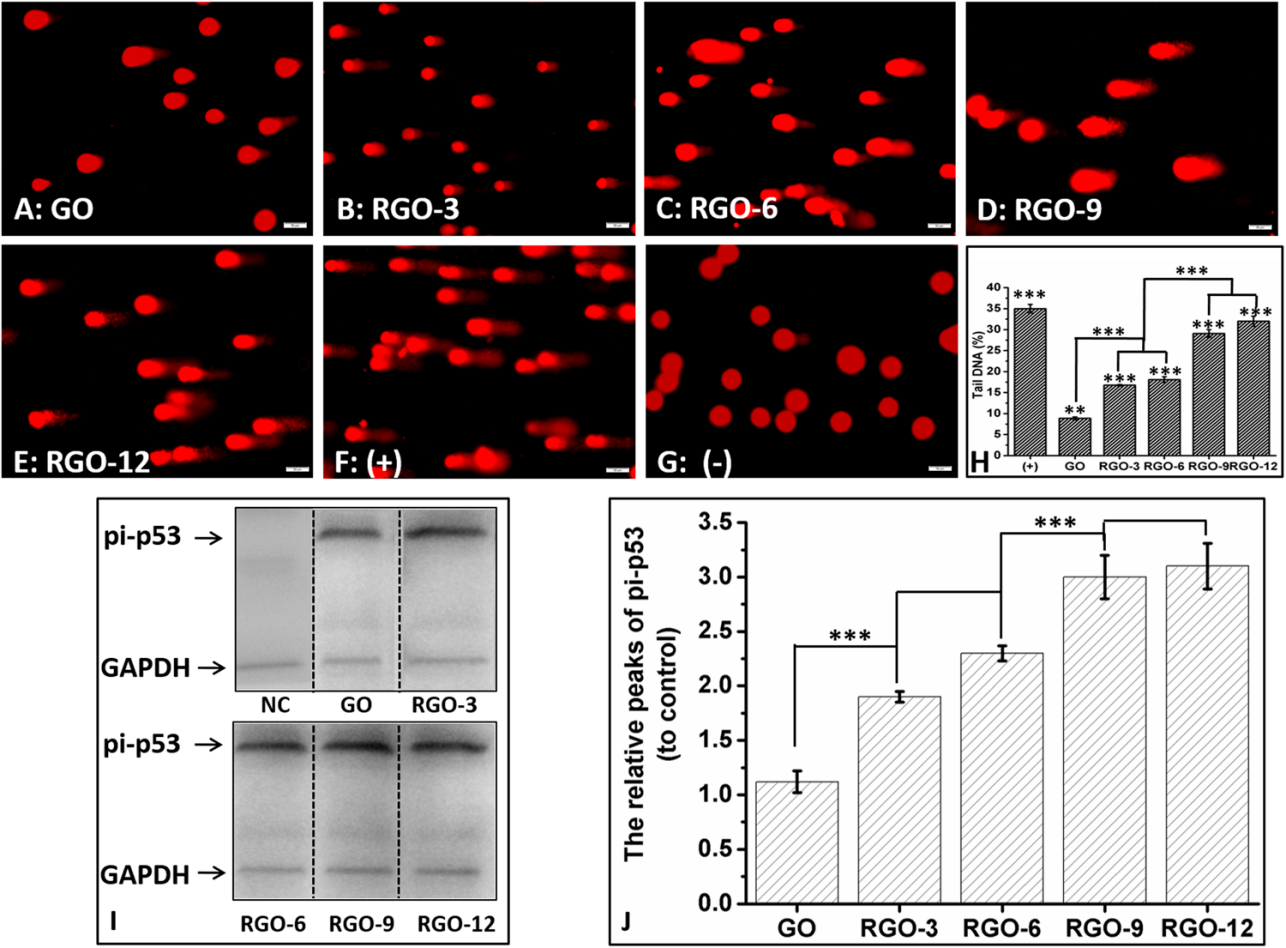
Fluorescence images of ARPE-19 cells during the Comet test after exposed to **A** GO, **B** RGO-3, **C** RGO-6, **D** RGO-9, **E** RGO-12, **F** the positive control, and **G** the negative control. Scale bar = 50 μm. **H** The percentages of tail DNA detected. **I** Western blot images of pi-p53 expression after exposure of ARPE-19 cells to GO or RGOs. **J** The relative amounts of pi-p53 to the control induced by GO or RGOs

DNA damage in of ARPE-19 cells induced by GO or RGOs after 24 h was assessed by the alkaline comet assay. Compared to the negative control (Fig. 5G), a 9% DNA damage tail was visible in the GO-incorporated sample (Fig. 5A), suggesting that GO caused DNA damage in ARPE-19 cells. Significant DNA damage tails (16–18%) were obtained when RGO-3 or RGO-6 was introduced (Fig. 5B, C), confirming that removal of oxygen-containing functional groups significantly increased the genotoxicity of GO. Moreover, ~30% DNA damage tails were observed when the RGO-9 or RGO-12 was introduced to the cells (Fig. 5D, E), which was comparable to the positive control (35% DNA tails) (Fig. 5f). The comet results suggested that both GO and RGOs induced the DNA damage in ARPE-19 cells after incubation for 24 h. However, much greater amounts of DNA damage were observed for the RGOs, indicating that RGOs prepared from biocompatible reduction processes induced much more DNA damage than GO. Interestingly, we have demonstrated elsewhere that hydroxyl-functionalized graphene (G-OH) showed less than 5% DNA damage tails in the comet test, even at the concentration of 100 μg/mL [7]. Comparing with the 9% tails obtained with GO, GOH exhibited much lower genotoxicity. GO contains the unsaturated carbon–oxygen double bonds (C = O) that are not present in GOH. Das hypothesized that the reactive functional groups of GO interact with the macromolecules in cells and enhance the cytotoxicity [12]. However, on the basis of our results, we speculate that the increased DNA damage of GO-treated cells might be attributed to the presence of unsaturated C = O bonds, which are similar to some polyunsaturated lipids, facilitating the oxidation process and inducing further oxidative stress, which causes worse DNA damage [18]. However, the RGOs, especially RGO-6, RGO-9, and RGO-12, lost not only most of the C = O (Fig. 1, XPS) but also a great amount of saturated C–O bonds, which might enhance the compatibility of GO. Therefore, RGOs induced a significant ROS increase in the cells (Fig. 4F, ROS). In the report of Zhang et al. [13], the lower oxidation degree of GOs resulted in stronger indirect oxidative damage by promoting the conversion of H_2_O_2_ into ^•^OH. The direct oxidative abilities on cells of RGOs induced the DNA damage and finally decreased the cell viability.

DNA damage of GO or RGOs was further measured by the expression level of pi-p53 via Western blotting. pi-p53 is a DNA repair protein that remains inactive and has a short life time under normal conditions. p53 is activated by phosphorylation (pi-p53) when DNA is damaged. pi-p53 is stable and acts as a master guardian by triggering cell cycle arrest to provide time for the DNA repair [19]. Thus, the expression of pi-p53 is generally increased during the process of DNA damage. Our results clearly showed that the pi-p53 protein expression level increased after ARPE-19 cells were exposed to GO or RGOs (Fig. 5I). However, the expression of pi-p53 in RGO-treated cells was significantly increased compared to that in GO-treated cells. Furthermore, RGO-9- or RGO-12- treated cells expressed nearly 1.5 times more pi-p53 than the RGO-3- or RGO-6-treated cells (Fig. 5J). There was little statistical difference between RGOS-3 and RGO-6 or between RGO-9 and RGO-12. Western blot results further confirmed that both GO and RGOs caused DNA damage of ARPE-19 cells, while much more DNA damage was induced by the RGOs. These results were consistent with the comet results.

## Discussion

Our preliminary results revealed detailed studies on the genotoxicity of GO and RGOs. For better biocompatibility results, RGOs with different oxygen contents were prepared by using biocompatible Na-citrate as the reductant. Successful removal of oxygen-containing functional groups from GO to RGOs was confirmed by UV–Vis, FTIR, and XPS measurements. The strong cytotoxicity of RGOs and mild cytotoxicity of GO were confirmed by a cell viability assay. Both GO- and RGOs-induced DNA damage was observed in the comet test and expression of pi-p53. Particularly worse DNA damage was induced by GO than by GOH, suggesting possible roles of unsaturated C = O in introducing DNA damage. Although both GO and RGOs displayed ROS-dependant genotoxicity, higher expression of pi-p53 was indicative of more serious DNA damage, which was obtained in the cells exposed to RGOs. The ROS generation test confirms that even small amounts of GO and RGOs can induce DNA damage, but GO shows much less genotoxicity than the RGOs, suggesting that saturated C–O bonds are beneficial for better biocompatibility of GO. Therefore the oxygen-containing functional groups play essential roles in the genotoxicity of GBMs, in which the unsaturated C = O bonds and saturated C–O bonds might be attributed to opposite functions. Our results provide useful information for safe contact with GBMs and the potential for reducing the toxicity of GBMs via chemical modification.

## Supplementary information

Supplementary figure S1

Supplementary figure S2
