# Correction to: Oxygen content-related DNA damage of graphene oxide on human retinal pigment epithelium cells

**DOI:** 10.1007/s10856-021-06634-3

**Published:** 2022-02-09

**Authors:** Liling Ou, Xiujuan Lv, Zixia Wu, Weibo Xia, Yida Huang, Luya Chen, Wenjie Sun, Yao Qi, Mei Yang, Lei Qi

**Affiliations:** 1 State Key Laboratory of Ophthalmology, Optometry and Visual Science, Institute of Advanced Materials for Nano-Bio Applications, School of Ophthalmology and Optometry, School of Biomedical Engineering, Wenzhou Medical University, Wenzhou, China; 2 Department of Ultrasonic, The First Hospital of Qiqihar, Affiliated Qiqihar Hospital, Southern Medical University, Qiqihar, China

Correction to: *Journal of Materials Science: Materials in Medicine* (2021) 32:20

10.1007/s10856-021-06491-0 published online 27 February 2021

The authors agree to withdraw the Fig. 5J and replace Fig. 5 as given below. In addition, on page 8, left column, the last four lines “However, the expression of pi-p53 in RGO-treated cells was significantly increased compared to that in GO-treated cells. Furthermore, RGO-9- or RGO-12- treated cells expressed nearly 1.5 times more pi-p53 than the RGO-3- or RGO-6-treated cells (Fig. 5J). There was little statistical difference between RGOS-3 and RGO-6 or between RGO-9 and RGO-12.” should read as “However, the expression of pi-p53 in RGO-treated cells was increased compared to that in GO-treated cells”.

**Fig. 5:**
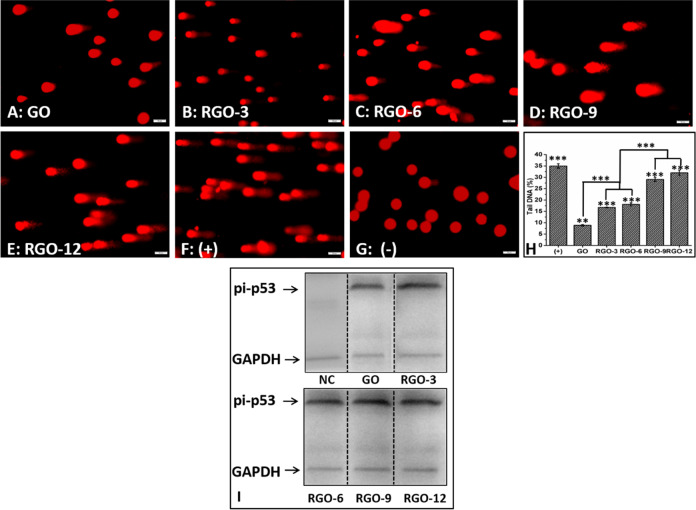
Fluorescence images of ARPE-19 cells during the Comet test after exposed to **A** GO, **B** RGO-3, **C** RGO-6, **D** RGO-9, **E** RGO-12, **F** the positive control, and **G** the negative control. Scale bar = 50 μm. **H** The percentages of tail DNA detected. **I** Western blot images of pi-p53 expression after exposure of ARPE-19 cells to GO or RGOs

## Supplementary information

Supporting Information-2

