# Supplementary figures and images for: Oxygen content-related DNA damage of graphene oxide on human retinal pigment epithelium cells
Source: J Mater Sci Mater Med. 2021 Feb 27;32(2):20. doi: 10.1007/s10856-021-06491-0 (PMC7914238; doi:10.1007/s10856-021-06491-0)

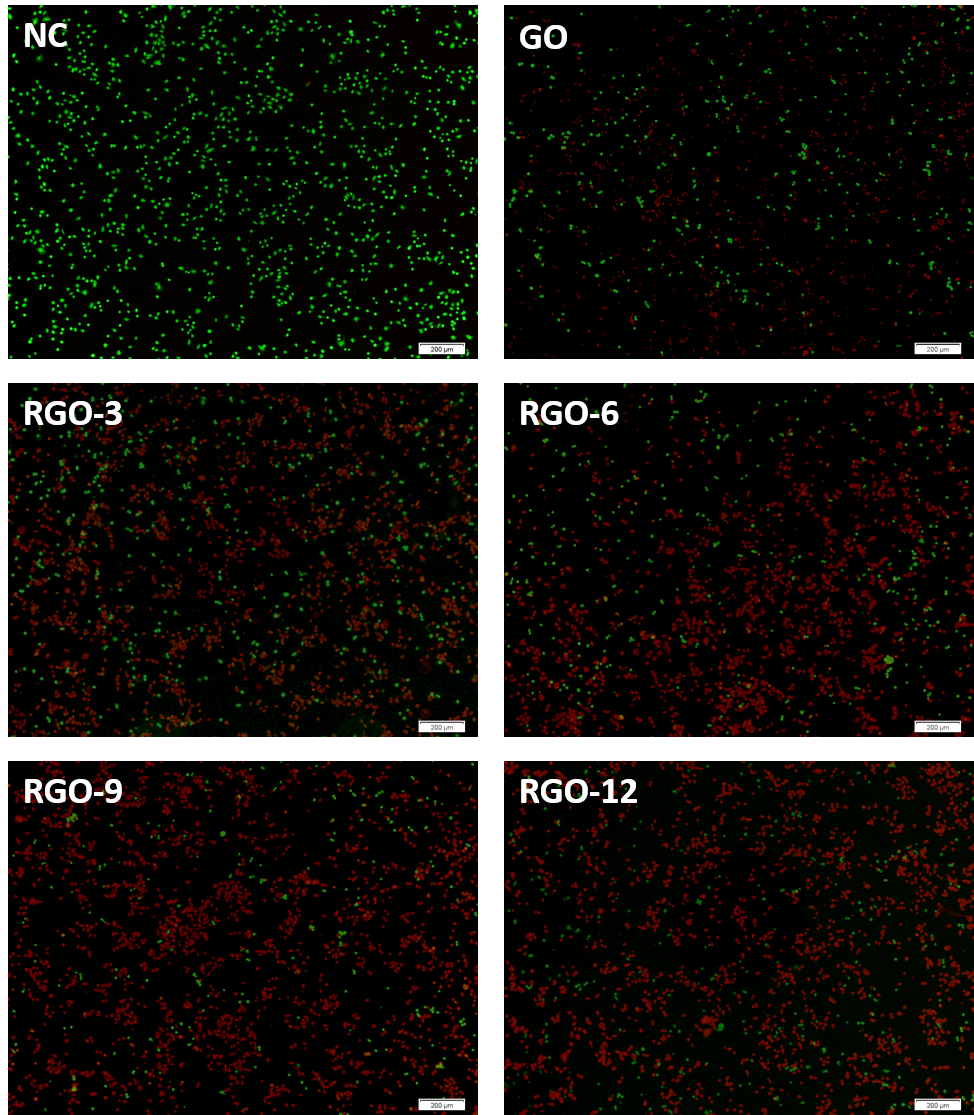

Supplement: Supplementary file 1 — Supplementary figure S1 [file 10856_2021_6491_MOESM1_ESM.tif]

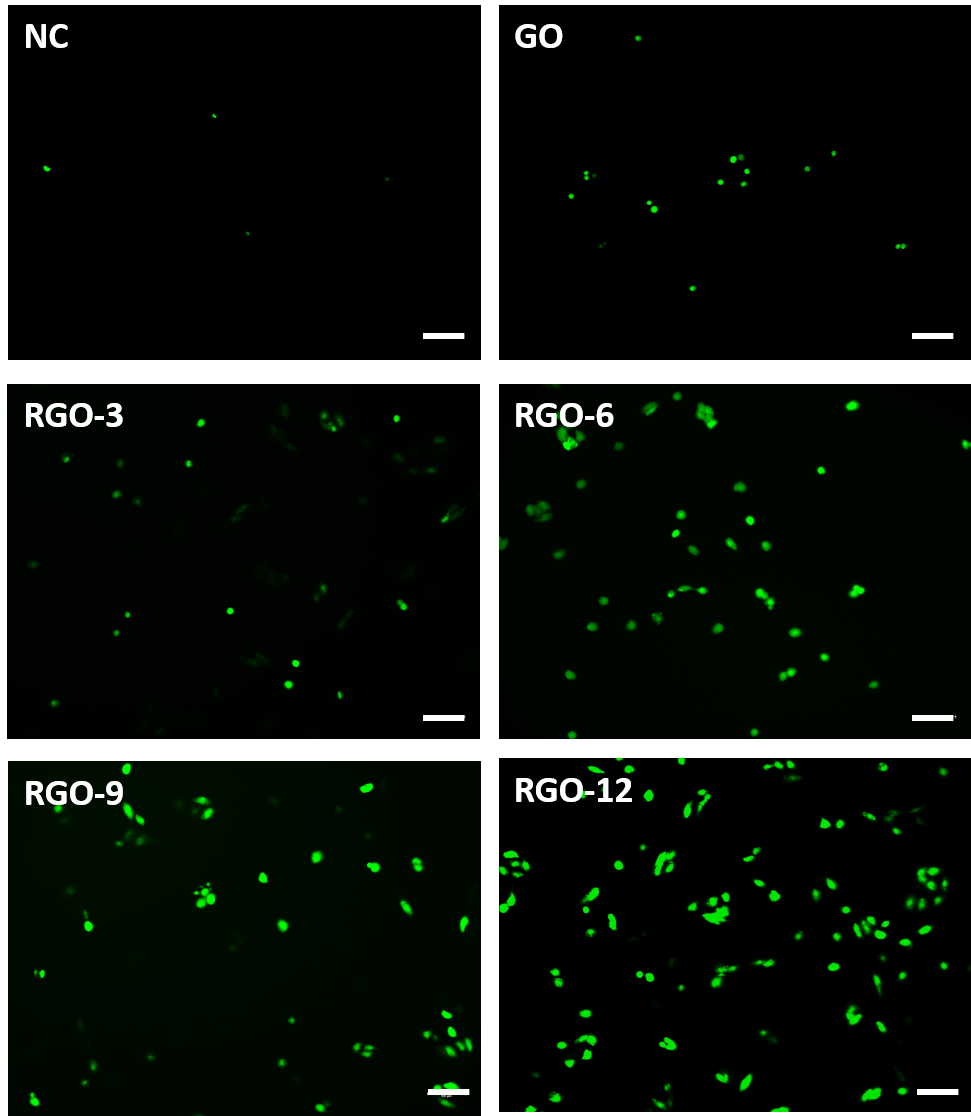

Supplement: Supplementary file 2 — Supplementary figure S2 [file 10856_2021_6491_MOESM2_ESM.tif]
