# Supplementary material for: Correction to: Oxygen content-related DNA damage of graphene oxide on human retinal pigment epithelium cells
Source: J Mater Sci Mater Med. 2022 Feb 9;33(2):23. doi: 10.1007/s10856-021-06634-3 (PMC8828594; doi:10.1007/s10856-021-06634-3)

**Supporting Information-2**

**Oxygen Content Related DNA Damage of Graphene Oxide on Human** **Retinal Pigment Epithelium Cells**

**Running title: DNA damage of Graphene oxide**

Liling Ou^1¶^, Xiujuan Lv^1¶^, Zixia Wu^1¶^, Weibo Xia^1^, Yida Huang^1^, Luya Chen^1^, Wenjie Sun^1^, Yao Qi^2*^, Mei Yang^1*^, Lei Qi^1*^

^1^State key Laboratory of Ophthalmology, Optometry and Visual Science, Institute of Advanced Materials for Nano-Bio Applications, School of Ophthalmology and Optometry, School of Biomedical Engineering, Wenzhou Medical University, 270 Xueyuanxi Road, Wenzhou 325027, China

^2^Department of Ultrasonic, The First Hospital of Qiqihar, Affiliated Qiqihar Hospital, Southern Medical University, Qiqihar 161005, China

^¶^These authors contributed equally to this work

***Corresponding Authors**

Yao Qi, Mei Yang, Lei Qi

E-mail: [qiyao914@163.com](mailto:qiyao914@163.com); [meiyang112686@wmu.edu.cn](mailto:meiyang112686@wmu.edu.cn); [imdoll@163.com](mailto:imdoll@163.com) (QL)

Tel: +86-18072196518

Fax: +86-0577-88067973

1. Supplementary statement about Fig.3: Significance was calculated between the GO-treated or RGOs-treated groups versus the negative control group (NC); the symbol of “” acts as a “brace”, the groups within it have the same significance versus NC group.
2. 1) The original figure of fig.5I (A. the original figure of fig.5I including the RGO-3, GO, RGO-6, RGO-9 and RGO-12 groups; B. the marker information of figure A)


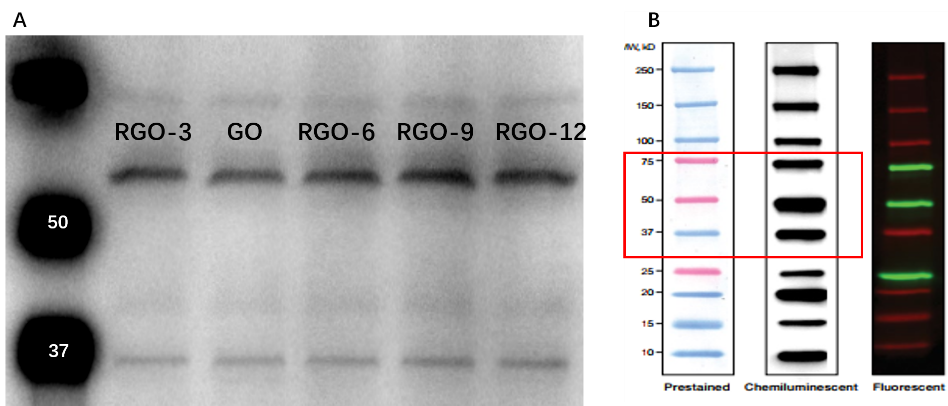


2) The original figure of fig.5I (the negative control group, which was circled in the figure)


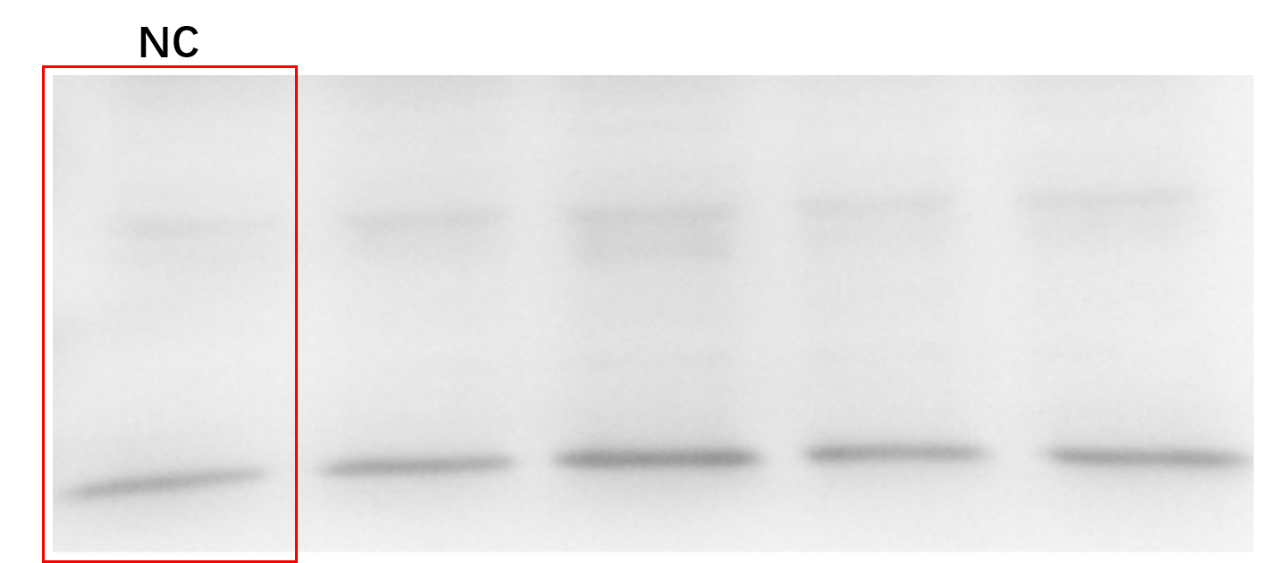

Supplement: Supplementary file 1 — Supporting Information-2 [file 10856_2021_6634_MOESM1_ESM.docx]
